# 1722. Impact of Universal Conjugate Pneumococcal Vaccination in Infants on the Incidence and Mortality of Pneumococcal Meningitis in the General Population in Uruguay, South America (2005 – 2022).

**DOI:** 10.1093/ofid/ofad500.1554

**Published:** 2023-11-27

**Authors:** Marcos Delfino, Monica Pujadas Ferrer, María Catalina Pírez

**Affiliations:** Faculty of Medicin, University of the Republic, Montevideo, Montevideo, Uruguay; Faculty of Medicine University of the Republic Uruguay, Montevideo, Montevideo, Uruguay; Faculty of Medicin, University of the Republic, Montevideo, Montevideo, Uruguay

## Abstract

**Background:**

Universal vaccination of children with conjugate vaccines was introduced using the 7 – valent pneumococcal conjugate vaccine (PCV7), which was changed to PCV13 (2 + 1 schedule, years 2008 to 2010), with nationwide coverage of 95 %.

The objective of this study is to analyze the impact of universal children vaccination with PCV7 and PCV13 on the incidence and mortality of pneumococcal meningitis (PM) in the general population in Uruguay.

**Methods:**

This is a descriptive, retrospective study that includes all cases (children and adults) reported to the Department of Health Surveillance of the Ministry of Public Health with a diagnosis of PM between January 1, 2005, and December 31, 2022. Variables: cases per year, sex, age (≤ 15 years and > 15 years), identified serotype, and deaths by year. The average rates of cases per 100,000 inhabitants are described in two periods: Period 1 or pre – universal vaccination (P1) from 2005 to 2007 and Period 2 or post – universal vaccination (P2) from 2011 to 2022, with their respective confidence intervals. Statistical analysis were established based on frequency distribution and statistical significance tests as appropriate, considering a value of *p* equal to or less than 0.05 as statistically significant. The research was approved by the institutional Ethics Committee.

**Results:**

The total number of cases (N) is 555, 56.5% are men. 102 cases from the vaccine introduction period (2008 to 2010) were excluded. The N for P1 is 149 (3 years), while for P2 it is 304 (12 years).

The main results of incidence and mortality are shown in Table 1 and in the attached graphs. Lethality remains high (26.5% average for the total period).

The vaccine serotypes (VS) decrease from 28.2% (95% CI: 20 to 35) in P1 to 12.2% (95% CI: 8.4 to 15) in P2, p: 0.00006. The non – VS increase from 18.1% (95% CI: 11 to 24) in P1 to 44.7% (95% CI: 39.1 to 50.3) in P2, p: 0.00000001.

The predominant VS in P1 is 14 (26.2%, 95% CI: 13-39); in P2 there are 8.1%, 95% CI: 0.7-16.9), p: 0.071. The predominant VS in P2 is 3 (46%, 95% CI: 30-62); in P1 9.5% (95% CI: 0.6-18), p: 0.0003.

The non – VS predominant in both periods is 12F.

**Figure:**
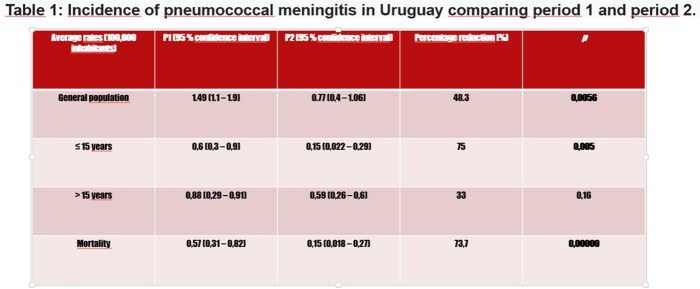

Evolution of incidence rates of PM in the general population and in people under and over 15 years of age as a function of time (in years).

**Figure:**
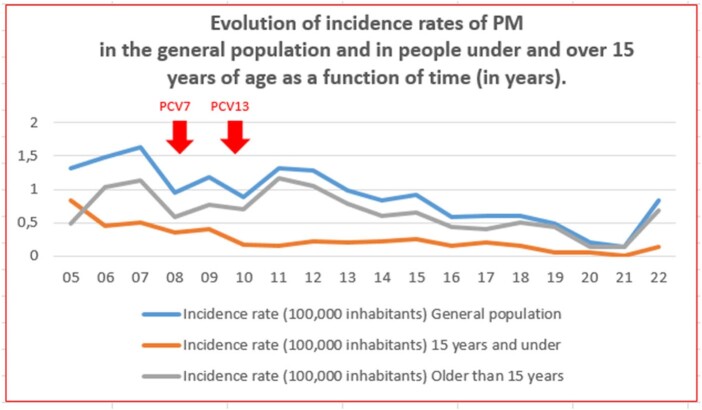

Mortality rates

**Figure:**
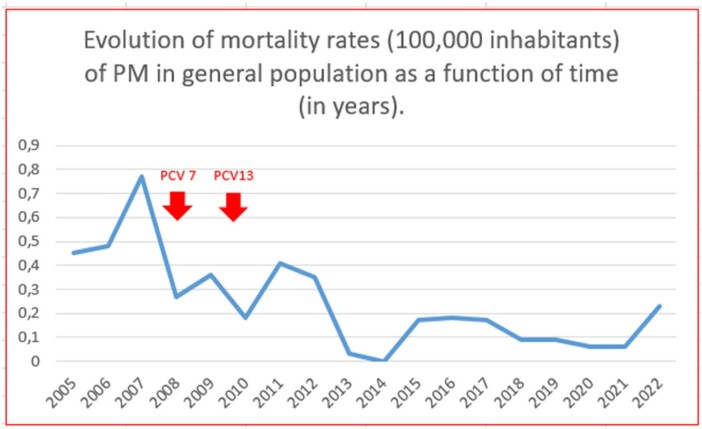

**Conclusion:**

The incidence and mortality of PM statistically decreased in children under 15 years old and in the general population, which can be attributed to herd immunity.

**Disclosures:**

**Marcos Delfino, Pediatrician, Pediatric Infectious Diseases**, Pfizer: Finna **María Catalina Pírez, Pediatrician, Pediatric infectologist, microbiologist Professor of pediatric, Degree V**, Merck, Pfizer: Expert Testimony|Merck, Pfizer: Honoraria

